# Adaptive DBSCAN Clustering and GASA Optimization for Underdetermined Mixing Matrix Estimation in Fault Diagnosis of Reciprocating Compressors

**DOI:** 10.3390/s24010167

**Published:** 2023-12-27

**Authors:** Yanyang Li, Jindong Wang, Haiyang Zhao, Chang Wang, Qi Shao

**Affiliations:** 1 College of Mechanical Science and Engineering, Northeast Petroleum University, Daqing 163318, China; liyanyang@byau.edu.cn (Y.L.); wjd327@126.com (J.W.); 15826930946@163.com (C.W.); 15145616172@163.com (Q.S.); 2 College of Civil Engineering and Water Conservation Institute, Heilongjiang Bayi Agricultural University, Daqing 163319, China

**Keywords:** underdetermined blind source separation, genetic simulation annealing algorithm, DBSCAN, reciprocating compressor

## Abstract

Underdetermined blind source separation (UBSS) has garnered significant attention in recent years due to its ability to separate source signals without prior knowledge, even when sensors are limited. To accurately estimate the mixed matrix, various clustering algorithms are typically employed to enhance the sparsity of the mixed matrix. Traditional clustering methods require prior knowledge of the number of direct signal sources, while modern artificial intelligence optimization algorithms are sensitive to outliers, which can affect accuracy. To address these challenges, we propose a novel approach called the Genetic Simulated Annealing Optimization (GASA) method with Adaptive Density-Based Spatial Clustering of Applications with Noise (DBSCAN) clustering as initialization, named the CYYM method. This approach incorporates two key components: an Adaptive DBSCAN to discard noise points and identify the number of source signals and GASA optimization for automatic cluster center determination. GASA combines the global spatial search capabilities of a genetic algorithm (GA) with the local search abilities of a simulated annealing algorithm (SA). Signal simulations and experimental analysis of compressor fault signals demonstrate that the CYYM method can accurately calculate the mixing matrix, facilitating successful source signal recovery. Subsequently, we analyze the recovered signals using the Refined Composite Multiscale Fuzzy Entropy (RCMFE), which, in turn, enables effective compressor connecting rod fault diagnosis. This research provides a promising approach for underdetermined source separation and offers practical applications in fault diagnosis and other fields.

## 1. Introduction

Machinery fault diagnosis plays a pivotal role in the industrial sector, particularly in high-temperature and high-pressure working environments, where early fault detection can prevent catastrophic accidents resulting from component failures [1]. Signal processing is a key tool for achieving early fault detection, with a specific focus on vibration signals. However, field-collected signals often comprise a mixture of multiple sources, and the unpredictable nature of fault locations complicates the acquisition of clean data due to insufficient sensor coverage [2]. To address this challenge, the primary task at hand is to separate and reconstruct signals in cases where the number of signal sources is unknown, and the propagation channels are uncertain.

Blind Source Separation (BSS) is a signal separation technology inspired by the “cocktail party problem”. Depending on the number of source signals (n) and sensors (m), BSS models are categorized as overdetermined BSS (m>n), positive definite BSS (m=n), and underdetermined BSS (m<n). In recent years, underdetermined blind source separation (UBSS) has garnered significant attention due to its capacity to successfully separate source signals, even in cases where sensors are insufficient. Consequently, UBSS has found applications in diverse fields such as speech recognition [3,4], image processing [5], and biomedical engineering [6].

Sparse Component Analysis (SCA) is a classic underdetermined blind source separation technique that has undergone significant evolution within the field of signal processing. Initially rooted in the “source disjointness” assumption (WDO) [7], SCA operated under the premise that each observed point in the time–frequency domain corresponded exclusively to a single source. However, as research progressed, the source sparsity assumption was relaxed, enabling the simultaneous activity of multiple sources at the same time–frequency points [8,9,10]. This transition expanded the flexibility of SCA, making it more suitable for real-world scenarios where strict source disjointness may not apply. Furthermore, SCA has been extended to accommodate cases with more than two observations, broadening its applicability to situations involving a variety of observations and sources [11,12]. This adaptation enabled the application of SCA in situations involving a broader array of observations and sources. A noteworthy refinement was the transition from identifying single-source zone work s to pinpointing single-source points, and the present paper adopts this single-source point assumption [13]. SCA has found application not only in the realm of instantaneously mixed signals but also in scenarios such as anechoic environments, convolutive mixtures [14], and even (post-)nonlinear mixtures, e.g., [15,16,17]. SCA has also been integrated successfully with source localization techniques, enabling more accurate estimations of source numbers and locations. Some ad hoc clustering methods have been proposed to count and locate sources effectively [18]. Additionally, SCA methods bear similarities with Convex Non-Negative Matrix Factorization (NMF) and Volume-Constrained NMF, with ongoing efforts to extend their applicability to hyperspectral unmixing and audio domains [19,20]. Certain SCA methods have been adapted to address scenarios with missing data entries [21].

In addition to its signal processing applications, SCA methods have found utility in machinery and equipment fault diagnosis. Although the research in this area began relatively late, the collaborative efforts of experts have introduced SCA methods to the field of diagnosis [22]. For instance, Hu et al. [23] effectively employed sparse component analysis for underdetermined blind source separation in diagnosing wind turbine gearbox bearing faults. Hao et al. [24] introduced the use of the wavelet mode maximum and the potential function method, resulting in higher fault diagnosis accuracy compared to traditional SCA methods. He et al. [25] proposed pre-processing and whitening of observed signals based on traditional Sparse Component Analysis (SCA) to attenuate interference components, effectively using it for feature extraction in compressor blade fault detection. Wang et al. [26] introduced Refined Composite Multiscale Fuzzy Entropy (RCMFE) to explore hidden fault information in vibration signals and successfully realized fault feature extraction in reciprocating compressors.

The evolution of SCA continues to drive innovation in the field, making it a valuable and versatile tool in signal processing applications. It only requires the source signal to satisfy sparsity to separate it from the mixed signal [27,28,29,30]. Under the assumption of sparse signals, the estimation of the mixing matrix can be transformed into a clustering problem that is solved by a clustering algorithm. Traditional clustering algorithms, such as Fuzzy C-Means (FCM), require prior knowledge of the number of sources, making it less suitable for underdetermined scenarios [31,32]. To address this limitation, the DBSCAN method has been introduced to estimate the number of clustering centers, thereby overcoming the dependency on pre-determined source counts. However, setting initial parameters in DBSCAN can be challenging, requiring experience and affecting result accuracy [33,34].

Nonetheless, the Fuzzy C-Means (FCM) algorithm is a local search optimization method and can converge to local minima when initial values are not selected optimally. In response to this issue, researchers have integrated intelligent algorithms, such as simulated annealing and genetic algorithms, to enhance clustering performance [35,36,37]. Simulated annealing offers robust mathematical properties but suffers from slow convergence and parameter sensitivity. On the other hand, genetic algorithms provide a novel, evolution-based approach for solving complex problems. Integrating these algorithms with Fuzzy C-Means has been proposed to improve clustering performance [38,39,40].

In this context, our paper introduces the CYYM method, which leverages an adaptive DBSCAN algorithm and an improved GASA optimization algorithm to address the challenges associated with unknown source counts and noisy environments [41,42]. This method comprises two key steps: the adaptive DBSCAN method filters out noise points and determines the number of sources, while the GASA optimization algorithm automates clustering center identification and enhances matrix estimation accuracy with speed.

Our proposed algorithm offers several key advantages:

1. The adaptive DBSCAN method effectively filters noise and accurately identifies source numbers, facilitating precise matrix estimation.
2. The integration of the GASA optimization algorithm combines global exploration capabilities with local search, avoiding local optima and improving clustering center identification.
3. The optimized GASA algorithm provides sensible control parameter settings, enhancing search capabilities and evolution speed.
4. Leveraging the k-dist curve improves denoising and clustering, which are adaptively integrated into the adaptive DBSCAN algorithm.

In summary, our algorithm enhances clustering accuracy, automates center identification, provides sensible parameter settings, and significantly improves denoising and clustering. The rest of the paper is divided into five parts. Section 1 presents the basic theory. Section 2 introduces the adaptive DBSCAN, the GASA optimization, and the proposed method. The simulation analysis and the compression application are provided in Section 3. Section 4 contains the conclusion.

## 2. Basic Theory of Blind Source Separation

### 2.1. The Mathematical Model

Blind source separation applied to fault diagnosis needs to cope with the challenge of a large number of source signals S(t) (fault signals) passing through an unknown transmission system **A**, initially getting mixed, and subsequently, being received alongside noise signals N(t) by a limited number of sensors, resulting in observation signals X(t). Based on the above analysis, the mathematical model of the basic technique of blind source separation can be expressed as follows:

(1) X(t)=A×S(t)+N(t)

where X(t) $={\left[x_{1}\left(t\right),x_{2}\left(t\right),\ldots ,x_{m}\left(t\right)\right]}^{T}$ represents *m* observation signals collected by m sensors; S(t) $={\left[s_{1}\left(t\right),s_{2}\left(t\right),\ldots ,s_{n}\left(t\right)\right]}^{T}$ represents *n* statistically independent source signals, **A** represents the mixing matrix of the unknown m×n(m<n), and N(t) denotes a noise signal. Neglecting noise, writing (1) in matrix form, the model can be rewritten as follows:

(2) $$\left[\begin{array}{c} x_{1}\left(t\right)\\ x_{2}\left(t\right)\\ \vdots \\ x_{m}\left(t\right) \end{array}\right]=\left[\begin{array}{cccc} a_{11} & a_{12} & \cdots & a_{1n}\\ a_{21} & a_{22} & \cdots & a_{2n}\\ \vdots & \vdots & \ddots & \vdots \\ a_{m1} & a_{m2} & \cdots & a_{mn} \end{array}\right]\left[\begin{array}{c} s_{1}\left(t\right)\\ s_{2}\left(t\right)\\ \vdots \\ s_{n}\left(t\right) \end{array}\right]$$

In general, mechanical vibration signals are not sparse in the time domain. In this paper, the sparse representation is realized by the STFT, and the equation is as follows:

(3) $$X\left(t,\omega \right)=\int_{-\infty }^{\infty }x\left(\tau \right)\omega^{*}\left(\tau -t\right)e^{-j\omega \tau }d\left(\tau \right)$$

where w(τ−t) is a window function, * denotes complex conjugation, and x(τ) is an observation signal.

**Figure 1:**
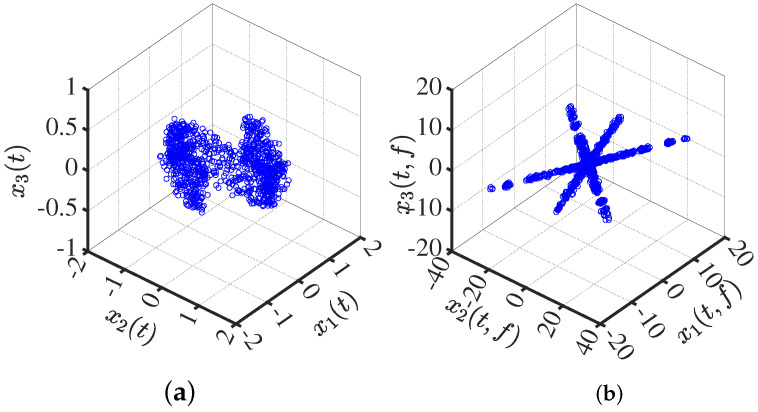
Mixed-signal scatter plot: (**a**) in the time domain; (**b**) in the time–frequency domain.

Transform the mixed signal into the sparse domain. In Figure 1, the real part of the time–frequency transforms is presented. It is evident that the transform in the time–frequency domain exhibits a certain sparsity, and its scatter plot reveals prominent linear characteristics.

### 2.2. Single-Source Point Detection

In Equation (2), assume that signal X(t) and signal S(t) are independent of each other. The sparsity of source signals means that only a few sources are active at a sampling time, and the amplitude of the rest of the sources approaches or equals zero. Suppose that, at the moment *i*, the source signal $s_{i}$ is activated, Equation (2) is expressed by selecting the single-source point of the signal at a certain time frequency as follows:

(4) $$\left[\begin{array}{c} x_{1}\left(t_{k},f_{k}\right)\\ x_{2}\left(t_{k},f_{k}\right)\\ \vdots \\ x_{m}(\left(t_{k},f_{k}\right) \end{array}\right]=\left[\begin{array}{c} a_{1i}\\ a_{2i}\\ \vdots \\ a_{mi} \end{array}\right]\left[\begin{array}{c} s_{i}\left(t_{k},f_{k}\right) \end{array}\right]$$

Furthermore, if Equation (4) is deformed, then Equation (5) is valid:

(5) $$\frac{x_{1}\left(k\right)}{a_{1i}}=\frac{x_{2}\left(k\right)}{a_{2i}}\cdots \frac{x_{m}\left(k\right)}{a_{mi}}=s_{i}\left(k\right)$$

Accordingly, the single-source point in the signal has a linear clustering characteristic. The general principle of single-source point is: in the time–frequency domain, the criterion is whether the difference is zero or not, which is between the ratio of the imaginary part and the real part of the observed signal $x_{i}\left(t_{k},f_{k}\right)$, $x_{j}\left(t_{k},f_{k}\right)$ at the same time–frequency point.

(6) $$\frac{I(x_{i}\left(t_{k},f_{k}\right))}{R(x_{i}\left(t_{k},f_{k}\right))}-\frac{I(x_{j}\left(t_{k},f_{k}\right))}{R(x_{j}\left(t_{k},f_{k}\right))}=0$$

Considering the noise, the threshold λ is relaxed; in general, λ is between 0 and 1:

(7) $$∥\frac{I(x_{i}\left(t_{k},f_{k}\right))}{R(x_{i}\left(t_{k},f_{k}\right))}-\frac{I(x_{j}\left(t_{k},f_{k}\right))}{R(x_{j}\left(t_{k},f_{k}\right))}∥<\lambda$$

**Figure 2:**
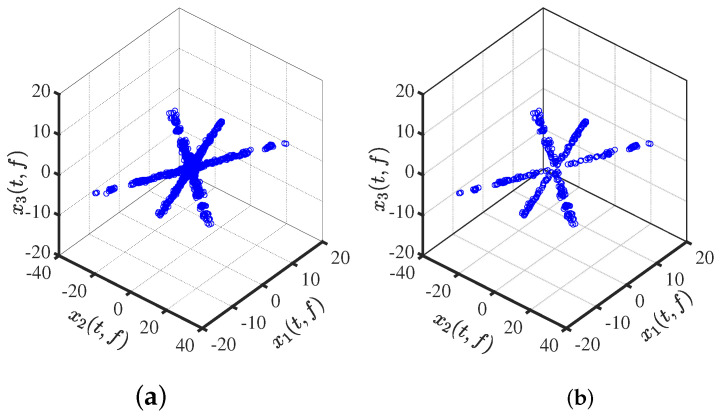
Time–frequency scatter plot: (**a**) After the elimination of low energy points. (**b**) After the detection of single-source points.

The single-source point vector is obtained, low-energy points (<0.1 times the average value) are excluded, and some low-energy noise points are eliminated for the accuracy of the mixture matrix estimation as shown in Figure 2a; the linear feature information of the mixed signal is retained. After removing the multisource points, the linear clustering property is further enhanced, as shown in Figure 2b.

Hence, the column vectors in the mixed matrix are deduced by the above two aspects, i.e., the direction of the linear clustering and the number of projective clusters. Namely, the number of projection clusters is the number of columns in the mixed matrix, and the direction of the column vector can be derived from the direction of the linear clusters.

## 3. Adaptive DBSCAN Clustering and GASA Optimization

The CYYM algorithm is based on adaptive DBSCAN and GASA algorithms. Each of the following will be described.

### 3.1. Adaptive DBSCAN Clustering

#### 3.1.1. DBSCAN

Density-based spatial noise clustering (DBSCAN) is a representative clustering algorithm in noisy data points. The core idea of DBSCAN is to find high-density data points in the data heap, search for nearby high-density data points using proximity search, and then connect the high-density data points into pieces to generate various shapes of data clusters [43]. The DBSCAN algorithm contains the following definition:

**Definition** **1.** *Eps is the neighborhood radius of the P data point: the distance between the point P and the collection of data points is less than Eps;*

**Definition** **2.** *The density of points P: the number of points in the Eps radius of the point P;*

**Definition** **3.** *Core Point: point P is defined as a core point (the MinPts threshold) with a density greater than that of MinPts; otherwise, marked as a non-core point;*

**Definition** **4.** *Boundary Point: when Q is not a core point, it is defined as a boundary point, but it belongs to the Eps neighborhood of the core point P;*

**Definition** **5.** *Noise Point: neither core point nor boundary point in the dataset;*

**Definition** **6.** *Direct Density Reachability: when P is the core point, the data Q are in the radius of the neighborhood P, and Q is the direct density reachable point of P.*

**Definition** **7.** *Density Reachable: point $P_{1}$, $P_{2}$, in the dataset $\left\{P_{1},P_{2}...P_{n}\right\}$, let $P_{1}=P,P_{n}=Q$; if $P_{i}$ and $P_{i+1}$ both are directly density reachable, then P and Q are density reachable points.*

**Definition** **8.** *Density-Connected: if the O point allows P and Q density-reached, then P and Q are density-connected points, and it is clear that density-linked is symmetric.*

In the clustering process of the DBSCAN algorithm, first select any data point P in the data D. If P is the core point and the Eps neighbor of P is not less than MinPts, then the Eps neighbor of *P* is chosen as the seed point, it is taken as the new core point, and the Eps neighbor of P is pulled in; thus, the clustering is extended until a set is generated. If P is a boundary point, the Eps neighbor of P has fewer data points than MinPts, and DBSCAN selects the next point in *D*. Noise points do not belong to any cluster of data points.

**Figure 3:**
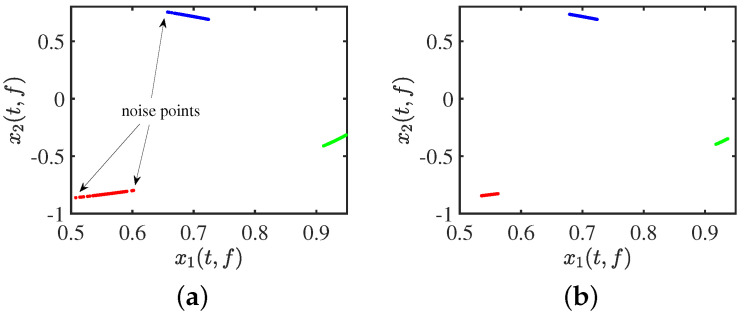
Clustering effect: (**a**) clustering by DBSCAN; (**b**) clustering by adaptive DBSCAN.

The effect of clustering using DBSCAN with an arbitrary input of initial parameters is shown in Figure 3a. After adjusting the parameters, we use DBSCAN to cluster the data, as is evident in Figure 3b. The clustering results are as we expected: compact between similar classes and distinct between dissimilar classes, with no noise points visible at all.

#### 3.1.2. ADBSCAN

**Figure 4:**
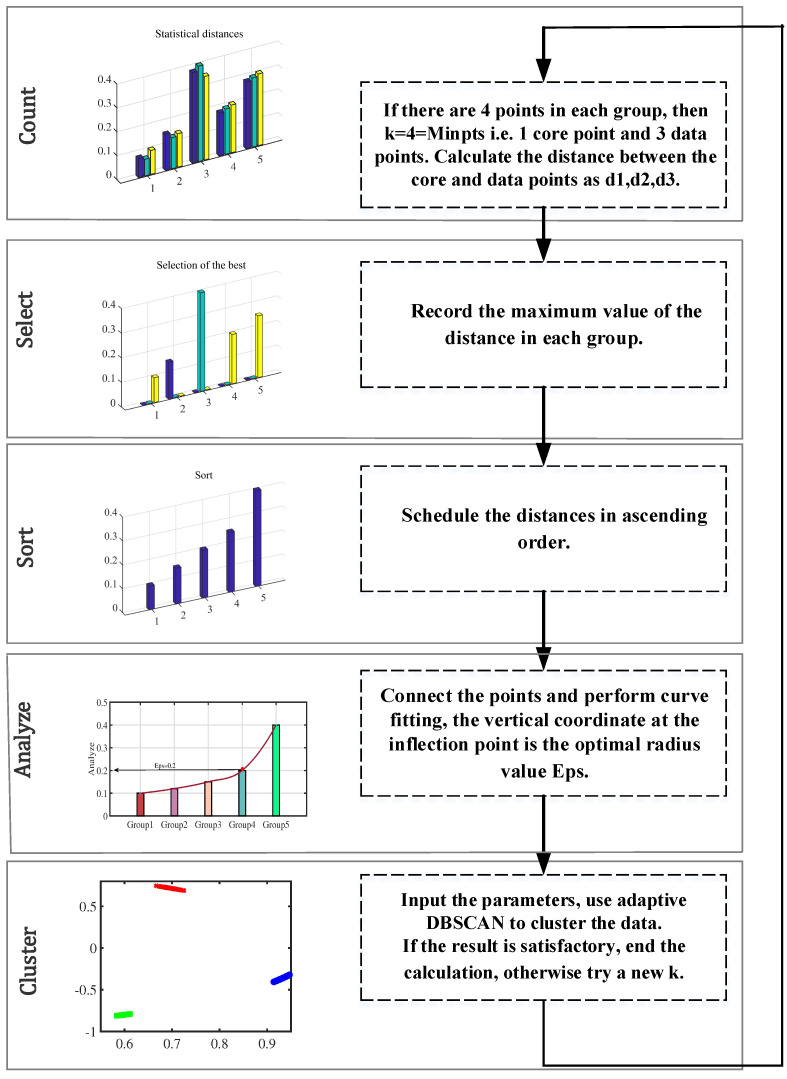
Process of adaptive DBSCAN clustering.

Users without prior knowledge are unable to identify the DBSCAN parameter setting regarding Eps and MinPts [44,45,46,47,48]. If the clustering radius (Eps) is too large, all the points will converge into one class, and the noise points cannot be eliminated effectively. If the clustering radius (Eps) is too small and the clusters increase enormously, then the computation of the whole process increases. The k-distribution (k−dist) curve is employed to establish the location of the inflection point and extract the parameter Eps. To illustrate the process, let us consider a hypothetical scenario with 20 data points. The procedure of adaptive DBSCAN (ADBSCAN) clustering is elaborated in Figure 4 under this illustrative example. Let k represent the value of MinPts, which signifies the number of points within the cluster. In practical applications, the value of k can be adjusted continuously until the desired result is achieved. It is recommended to set the initial value of k to be greater than or equal to the number of dimensions plus one [49,50,51].

Specific steps are as follows (see Algorithm 1):
**Algorithm 1** Adaptive DBSCAN Clustering**Input:** Noise Threshold, Initial k1. k_dist_sequence [xi] = calculate_k_dist(xi, k)2. sorted_k_dist = sort(k_dist_sequence)Eps = max(sorted_k_dist)3. inflection_point = find_inflection_point(sorted_k_dist)optimal_radius = sorted_k_dist[inflection_point]4. clusters = DBSCAN(data, Eps = optimal_radius, MinPts = k)num_noise_points = count_noise_points(clusters)5.**If** num_noise_points ≤ noise_threshold:end_calculation**else:**k = k + 1**return** step 1

### 3.2. Genetic Simulated Annealing Optimization

The purpose of genetic simulated annealing optimization is to obtain an initial solution by a genetic process, and then perform a simulated annealing search, so the local search and global search are completed alternately.

#### 3.2.1. Encoding Method

**Figure 5:**
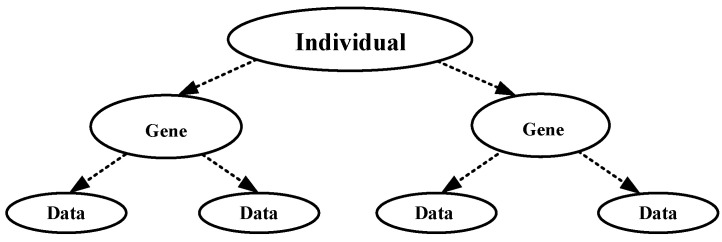
Tree coding structure.

The target of clustering is to aggregate disorganized data according to their similarity. Each cluster center is a table head with an arrow pointing to data belonging to that class. A tree structure is shown in Figure 5.

#### 3.2.2. Fitness Function

The search strategy of the genetic algorithm is to find the optimal solution using the fitness function as a criterion to evaluate the merits and demerits of individuals. Equations based on fuzzy clustering are as follows:

(8) $$J_{b}\left(U,v\right)=\sum_{k=1}^{n}\sum_{i=1}^{c}{\left(u_{ik}\right)}^{b}{\left(d_{ik}\right)}^{2}$$

(9) $$\left(d_{ik}\right)=d\left(x_{k}-v_{i}\right)={\left[\sum_{j=1}^{m}{\left(x_{kj}-v_{ij}\right)}^{2}\right]}^{1/2}$$

(10) $$u_{ik}=\frac{1}{\sum_{j=1}^{c}{\left(\frac{d_{ik}}{d_{jk}}\right)}^{\frac{2}{b-1}}}$$

(11) $$v_{ik}=\frac{\sum_{k=1}^{n}{\left(u_{ik}\right)}^{b}x_{kj}}{\sum_{k=1}^{n}{\left(u_{ik}\right)}^{b}}$$

where *U* is the similarity classification matrix, $d_{ik}$ is the Euclidean distance, $X=\left\{x_{1},x_{2},\ldots ,x_{n}\right\}$ refers to the data samples, $u_{ik}$ is the degree of membership in the class $A_{k}$, $\left\{v_{1},v_{2},\ldots ,v_{n}\right\}$ are cluster centers in each category, *b*(1<b<∞) is the weight coefficient, c(2≤c≤n) is the number of cluster centers, *n* is the number of samples, m is the number of feature samples, $f_{i}$ is the fitness, and $J_{b}=1⁄f_{i}$ is the lower the value of the function $J_{b}$, where the smaller the sum of the intraclass dispersion, the better the adaptability of the individual in the corresponding population.

#### 3.2.3. Select Operation

The fitness values are counted and sorted. The top 10% of the elite population is reproduced and inherited by future generations, while the rest is generated by roulette. Thus, the next generation can inherit good genes. The selection procedure is as follows.

Calculate the fitness function of individuals and the fitness of groups. $F=\sum_{i=1}^{n}f_{i}$, $p_{i}$ is the probability of selection of the individual, Equation (12), and $q_{i}$ is the cumulative value of the probability of selection, Equation (13). Randomly generate a number r in the range [0, 1]. If the condition $q_{1}>r$ is satisfied, $v_{1}$ is the first generation; otherwise, $v_{i}$(2<i<m) is the next generation on the condition of $q_{i}>r>q_{i-1}$.

(12) $$p_{i}=\frac{f_{i}}{F}\left(i=1,2.\ldots ,n\right)$$

(13) $$q_{i}=\sum_{j=1}^{i}p_{j}\left(i=1,2.\ldots ,n\right)$$

#### 3.2.4. Crossover Operator

**Figure 6:**
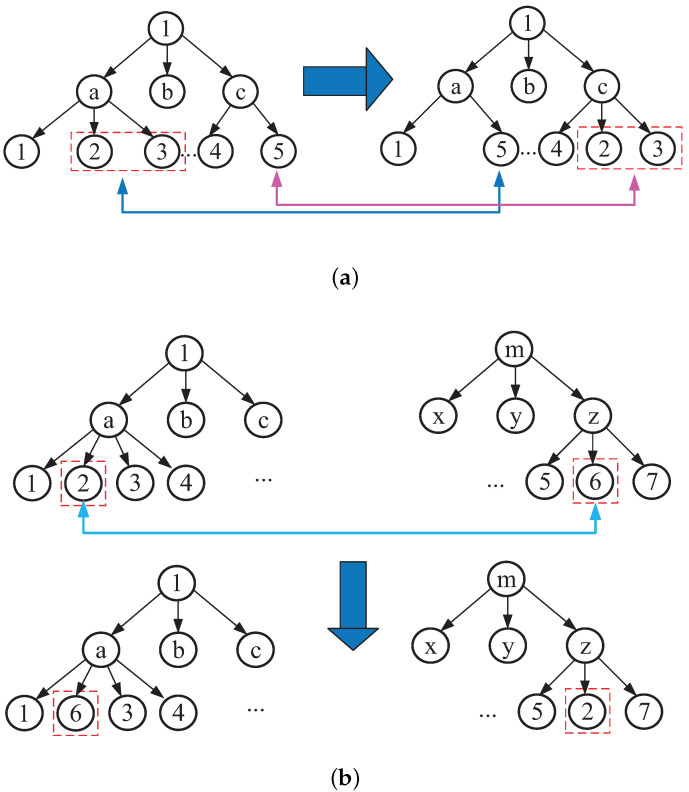
Two leaf nodes of a tree mutually exchanged: (**a**) same tree exchange; (**b**) different tree exchange.

New offspring are produced by replacing parts of the parent’s structure. In this process, the children choose their parents with equal probability. There are two kinds of crossover operators based on tree coding: one is to exchange two different leaf nodes with the same number of samples; the other is to exchange the leaf nodes from different trees. Figure 6a,b show the two crossover methods, respectively.

#### 3.2.5. Mutation Operation

To prevent premature convergence of the algorithm, the mutation operator is used to change the information in the leaf node, which maintains the diversity of genetics. The following procedure has been adopted: decimal numbers are generated randomly to select the tree for mutation, supposing the decimal number is less than the mutation rate, then leaf nodes are chosen randomly for conversion, and random numbers are generated which replace the leaf nodes.

#### 3.2.6. Individuals’ Simulated Annealing Operation

For newly created individuals, calculate the degree of membership using Equation (10), and calculate the cluster center using Equation (11). Simulated annealing algorithm to replace the old individual: if $f_{i}>f_{i}^{\prime }$, the new individual becomes the optimal solution, otherwise, it is accepted with a certain probability *P*:

(14) $$P=exp(\frac{f_{i}-f_{i}^{\prime }}{T})$$

where *T* is the control parameter and corresponds to temperature in thermodynamics, $f_{i}^{\prime }$ is the newly generated individual fitness, and $f_{i}$ is the old individual fitness.

#### 3.2.7. Conditions of Termination

Successive optimization is performed in step *Q* to achieve the final goal of the best individual in the population, with gen as the counting variable. If optimal, terminate and set gen=0, otherwise constantly optimize the index and change the cumulative count variable gen=gen+1. When gen=Q occurs, the updated population undergoes a new round of genetic and simulated annealing operations. When $T_{i}<T_{end}$, the calculation is terminated and the global optimal solution is obtained.

### 3.3. CYYM Algorithm Steps and Processes

For better clustering performance, the time–frequency points are transformed into compact clusters by normalization and assigned to the hypersphere in the upper right corner by mirror processing, as shown in Equation (15):

(15) $$\tilde{X}\left(t,f\right)=\frac{X(t,f)}{{∥X(t,f)∥}_{2}}\times_{\mathrm{sign}}\left(x_{1}\left(t,f\right)\right)$$

where the sign function is utilized to determine the sign of a number. It returns a value of 1 for positive numbers, 0 for zero, and −1 for negative numbers.

However, the specific number of clusters is not available on the time–frequency scatter plot at this point, and further cluster analysis is required. To identify the parameter setting of DBSCAN, we drew a k-dist curve and determined the position of the inflection point. The vertical scale of it (Eps) is the best value for the radius of the cluster, and the point whose distance exceeds Eps is regarded as the noise point. Based on it, the empirical parameters of DBSCAN are derived. Through the adaptive DBSCAN algorithm, the noise points are removed, and the number of clusters is obtained.

According to the steps of the genetic algorithm (GA), run the selection operator, select the crossover method for crossover operation, perform mutation operation, establish the evaluation mechanism, and select the advantage population to form the new species. Considering premature convergence, the periodic annealing process is added to GA, calling out the Metropolis sampling algorithm, and receiving poor individuals with a certain probability. When the genetic operation reaches a predetermined algebra, the optimal individual in the current population is taken as the initial solution of SA. With a decrease in temperature, the material energy tends to be stable. By reasonably setting the cooling schedule, the updated population undergoes a new round of genetic and simulated annealing operations. When $T_{i}<T_{end}$, the optimal global solution can be obtained.

**Figure 7:**
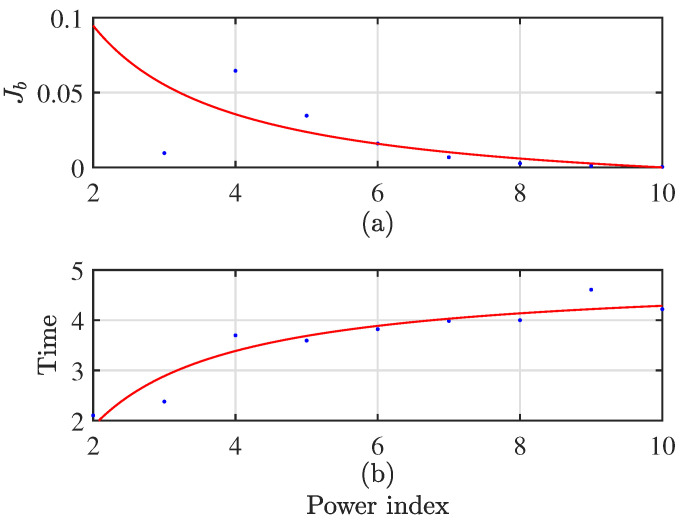
Weight coefficient decision diagram. (**a**) the trend graph of the fitness function as the power exponent increases; (**b**) the trend graph of computation time with an increasing power exponent.

**Figure 8:**
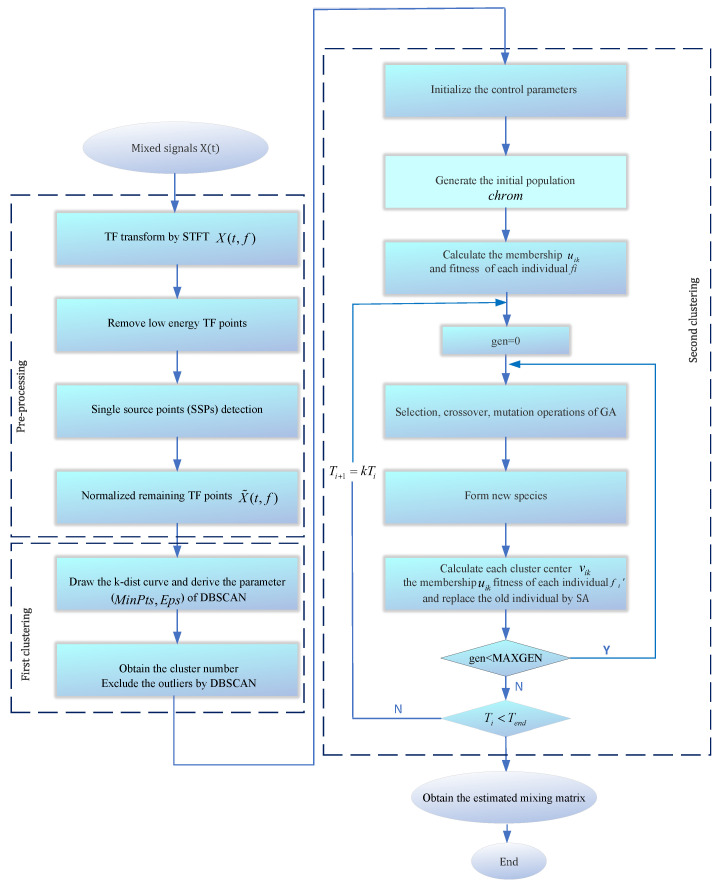
The flowchart of the CYYM method.

The selection of the improved GASA parameters is shown below: population size $p_{s}=10$, genetic algebra g=10, cross probability $p_{c}=0.7$, mutation probability $p_{m}=0.01$, initial annealing temperature $T_{0}=100$, terminal temperature $T_{end}=1$, and temperature cooling coefficient β=0.8. It is noteworthy that the choice of the weight coefficient *b* in fuzzy clustering using c-means is set to 6. The weight coefficient decision chart is illustrated in Figure 7. As the weight coefficient increases, $J_{b}$ decreases, leading to more desirable outcomes. Although there may be slight variations in computation time on each run, data collected according to statistical trends reveal that computation time tends to increase with a rising power index. Taking these trade-offs into consideration, setting the coefficient to 6 ensures excellent computational results with a relatively fast processing time. The flow diagram is depicted in Figure 8.

Specific steps of the CYYM are as follows (see Algorithm 2):
**Algorithm 2** CYYM Algorithm1.def signal_preprocessing(data):data = perform_STFT_conversion(data)data = perform_single_source_detection(data)data = remove_low_energy_points(data)data = normalize_spatial_mapping(data)return data2.def draw_k_dist_curve(data):k_dist_curve = calculate_k_dist_curve(data)inflection_point = locate_inflection_point(k_dist_curve)dbscan_params = derive_dbscan_parameters(inflection_point)return dbscan_params3.def dbscan_clustering(data, dbscan_params):clusters = run_dbscan(data, dbscan_params)return clusters4.Initialize Parameters for SApop_size = 10max_generations = 10crossover_prob = 0.7mutation_prob = 0.01initial_temperature = 100cooling_coefficient = 0.8termination_temperature =15.Initialize SA Algorithmcluster_centers = get_cluster_centers(clusters)population = initialize_population(pop_size, cluster_centers)compute_membership_and_fitness(population, data)6.Initialize Loop Countgeneration = 07.Genetic Operations**while** generation < max_generations:selected_population = select_population(population)offspring = crossover_and_mutation(selected_populationcrossover_prob, mutation_prob)new_population = form_new_population(population, offspring)compute_membership_and_fitness(new_population, data)8.Update Generationgeneration += 19.SimulatedAnnealingupdate_with_simulated_annealing(new_population, population, temperature)10.Check Termination**If** temperature < termination_temperature:return global_optimal_solution**else** repeat Genetic Operations11. mixing_matrix = estimate_mixing_matrix(cluster_centers)12. recovered_signals = recover_source_signals(data, mixing_matrix)

## 4. The Simulation Analysis and Compression Application

### 4.1. Evaluation of Indicators

The accuracy of the estimated mixed matrix is evaluated using normalized mean square error (*NMSE*) and deviation angle as criteria of interest. The *NMSE* expression is as follows:

(16) $$NMSE=10{\mathrm{lg}}_{}\left(\frac{\sum_{i=1}^{m}\sum_{j=1}^{n}{\left({\hat{a}}_{ij}-a_{ij}\right)}^{2}}{\sum_{i=1}^{m}\sum_{j=1}^{n}a_{ij}^{2})}\right)$$

where *m* and *n* denote, respectively, the rows and columns of the mixed matrix number, while ${\hat{a}}_{ij}$ and $a_{ij}$ represent, respectively, the elements in the *i*-th row and the *j*-th column of the estimated mixed matrix and the original mixed matrix. The *NMSE* value is used as a metric to assess the accuracy of the estimated matrix, where the smaller value indicates a more accurate estimation.

The expression of the deviation is the following:

(17) $$ang\left(a,\hat{a}\right)=\frac{180}{\pi }\arccos\left(\frac{\langle a,\hat{a}\rangle }{∥a∥\cdot ∥\hat{a}∥}\right)$$

where the deviation angle between the column vectors of $\hat{A}$ and A is represented, where $\hat{a}$ and *a* represent the column vector of $\hat{A}$ and A, respectively. A smaller deviation angle indicates a higher accuracy of the estimation matrix.

To further evaluate the similarity of the separated and source signals, the correlation coefficient is introduced. The larger the correlation coefficient, the more similar the recovered signal is to the source signal. The *SIR* serves as an indicator of the quality of a received signal. A higher *SIR* value signifies a more favorable signal quality, as it implies that the desired signal is significantly stronger in comparison to interference. Conversely, a lower *SIR* indicates that the received signal may be heavily affected by interference, which are calculated as follows:

(18) $$C=\frac{\sum_{k=1}^{K}\left|s_{i}\left(k\right){\hat{s}}_{i}\left(k\right)\right|}{\sqrt{\sum_{k=1}^{K}s_{i}^{2}\left(k\right)\sum_{k=1}^{K}{\hat{s}}_{i}^{2}\left(k\right))}}$$

(19) $$SIR=10lg[\frac{\sum_{k=1}^{K}s_{i}^{2}\left(k\right)}{\sqrt{\sum_{k=1}^{K}{\left(s_{i}\left(k\right)-{\hat{s}}_{i}\left(k\right)\right)}^{2}}}]$$

where $s_{i}\left(k\right)$ and ${\hat{s}}_{i}\left(k\right)$ represent the actual value and the estimated value of the second source signal, respectively, and *K* represents the length of time of the source signal on path *i*, that is, the number of sampling points of the source signal.

### 4.2. Experiment 1: Comparative Analysis of Accuracy in Mixed Matrix Estimation

**Figure 9:**
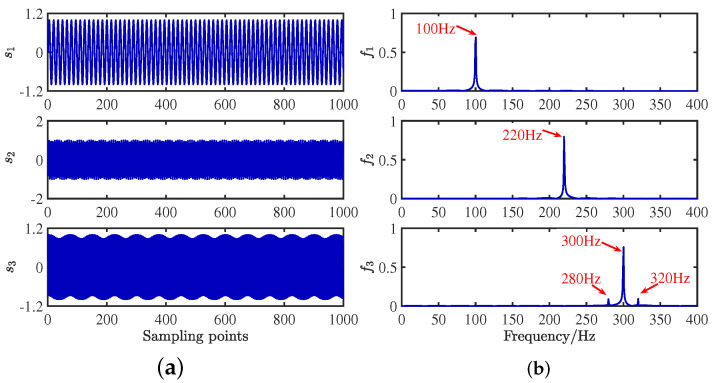
Waveforms of source signals: (**a**) in the time domain. (**b**) in the frequency domain.

To verify the feasibility of the CYYM method, three different mechanical vibration signals $S={\left[s_{1},s_{2},s_{3}\right]}^{T}$ are mixed and then separated by the CYYM method through simulation experiments. To be specific, $s_{1}$ is a sine signal, $s_{2}$ is a cosine signal, and $s_{3}$ is a frequency-modulated signal, shown in Equation (20). The sampling frequency is f = 1000 Hz, and N = 1024, which is the number of sample points. The time and frequency domain diagrams are depicted in Figure 9.

(20) $$\left\{\begin{array}{c} s_{1}=\sin\left(2\pi f_{1}t\right)\\ s_{2}=0.7\cos\left(2\pi f_{2}t+10\right)\\ s_{3}=\sin\left[\left(2\pi f_{3}t\right)+0.2*\cos\left(2\pi f_{m}t\right)\right] \end{array}\right.$$

where $f_{1}$ = 100 Hz, $f_{2}$ = 220 Hz, $f_{3}$ = 300 Hz, and $f_{m}$ = 20 Hz. In MATLAB, a random matrix A is generated by the function generator, and the matrix is normalized as shown in Equation (21):

(21) $$A=\left[\begin{array}{ccc} 0.6986 & 0.5575 & 0.9295\\ 0.7155 & -0.8301 & -0.3688 \end{array}\right]$$

Gaussian white noise with a mean of 0 and a variance of 0.1 is added to the mixed signal X(t) to simulate real environmental noise, as shown in Equation (22):

(22) X(t)=A×S(t)+N(t)

**Figure 10:**
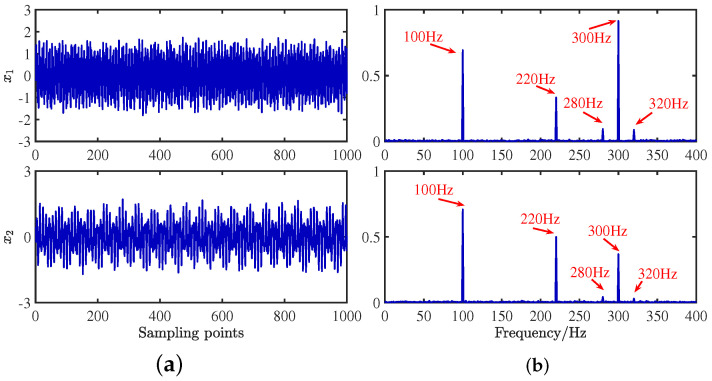
Mixed signals: (**a**) time domain waveforms; (**b**) envelope spectra.

The mixing waveforms are shown in Figure 10. It is discovered that the time–domain waveform features of the source signal are entirely submerged in the mixed signal. Meanwhile, in the corresponding spectrum, the characteristic frequencies of each source signal interfere with each other, and the characteristic frequencies of 280 Hz and 320 Hz are swamped by different frequencies, which demonstrates that the traditional frequency domain analysis method has some defects in dealing with mixed signals from multiple sources.

**Figure 11:**
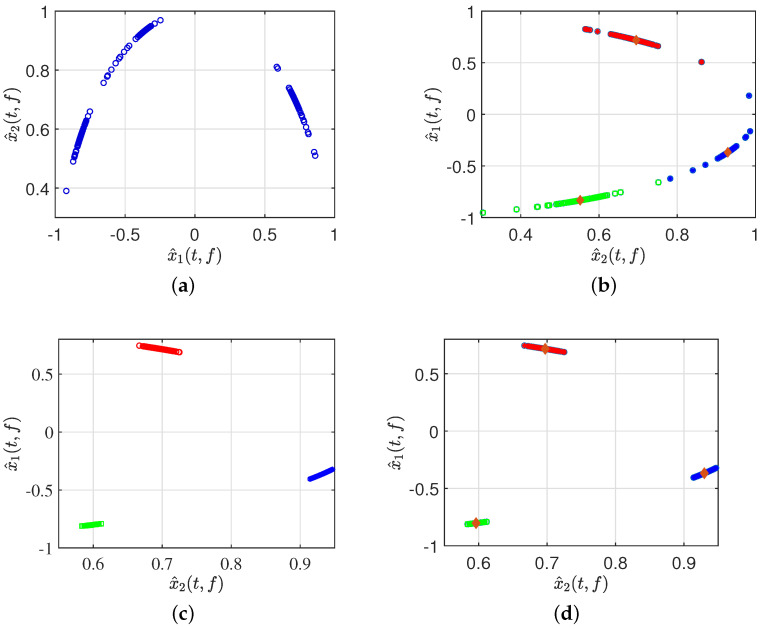
(**a**) Normalized time–frequency scatterplots. (**b**) Clusted by GASA. (**c**) Clusted by improved DBSCAN. (**d**) Clusted by CYYM.

Second, after signal pre-processing, three simulations were performed in Figure 11, specifically displaying the real part of the time–frequency transforms using different algorithms to validate the effectiveness of the proposed method. The scatter plot in Figure 11b represents the results clustered by GASA. The identification of the classification effect is observed to be low due to the presence of a significant number of outliers, greatly reducing the clustering accuracy of the clustering center. Our comparison of the GASA and CYYM algorithms for clustering revealed that the GASA algorithm is exceptionally sensitive to outliers. Consequently, the accurate estimation of the mixture matrix cannot be achieved by relying solely on the GASA method calculation.

As indicated in Figure 11c, three data stacks correspond to three source signals; moreover, with the help of adaptive DBSCAN preprocessing, the clustering in the first step provides a clear distinction between different groups and the expected effect of compactness in the same dataset, which provides a solid basis for further calculation of the location of the center of the cluster in the second step. The CYYM clustering is illustrated in Figure 11d. A GASA optimization algorithm was used based on Figure 11c to calculate the location of the cluster centers for each dataset and accurately label them to achieve the estimation of the UMM. There is a substantial increase in the speed of operation and a higher degree of computational accuracy.

The estimated value of ${\hat{A}}_{1}$ after applying the K-means algorithm to the normalized TF points is:

(23) $${\hat{A}}_{1}=\left[\begin{array}{ccc} 0.6930 & 0.5714 & 0.9261\\ 0.7200 & -0.8177 & -0.3735 \end{array}\right]$$

The estimated value of ${\hat{A}}_{2}$ after applying the DBSCAN algorithm to the normalized TF points is:

(24) $${\hat{A}}_{2}=\left[\begin{array}{ccc} 0.6889 & 0.5598 & 0.9284\\ 0.7142 & -0.8284 & -0.3715 \end{array}\right]$$

The estimated value of ${\hat{A}}_{3}$ after applying the GASA algorithm to the normalized TF points is:

(25) $${\hat{A}}_{3}=\left[\begin{array}{ccc} 0.6984 & 0.5575 & 0.9295\\ 0.7155 & -0.8297 & -0.3684 \end{array}\right]$$

The estimated value of ${\hat{A}}_{4}$ after applying the ADBSCAN algorithm to the normalized TF points is:

(26) $${\hat{A}}_{4}=\left[\begin{array}{ccc} 0.6984 & 0.5558 & 0.9293\\ 0.7155 & -0.8310 & -0.3689 \end{array}\right]$$

The estimated value of ${\hat{A}}_{5}$ after applying the FCM algorithm to the normalized TF points is:

(27) $${\hat{A}}_{5}=\left[\begin{array}{ccc} 0.6949 & 0.5614 & 0.9272\\ 0.7183 & -0.8264 & -0.3721 \end{array}\right]$$

The estimated value of ${\hat{A}}_{6}$ after applying the proposed method to the normalized TF points is:

(28) $${\hat{A}}_{6}=\left[\begin{array}{ccc} 0.6985 & 0.5574 & 0.9294\\ 0.7154 & -0.8299 & -0.3687 \end{array}\right]$$

To analyze and compare the estimation accuracy of the mixing matrices, the results of 100 simulation experiments using six different methods (K-means, FCM, DBSCAN, ADBSCAN, GASA, CYYM) were compared and analyzed based on the mean values of two metrics, NMSE (Normalized Mean Squared Error) and angular deviation, as shown in Table 1. The NMSE of the K-means algorithm is −38.4103 dB, indicating a relatively low accuracy, which may be attributed to a random selection of initial clustering centers. The GASA algorithm shows only a small enhancement compared to FCM, with an NMSE of −48.57108 dB. The NMSE obtained using the DBSCAN algorithm is −51.7364 dB, indicating a relatively good performance, though not yet reaching an optimal level. In contrast, ADBSCAN demonstrates a notable improvement in terms of the NMSE metric, achieving a value of −59.125, surpassing the performance of DBSCAN. The proposed CYYM method achieves an NMSE of −74.104 dB, which is the smallest value among all the methods. These results demonstrate that the clustering effect is more apparent and the precision is the highest when using the proposed CYYM method.

According to Table 1, the proposed method exhibits the smallest deviation angle, indicating the highest precision, followed by ADBSCAN, DBSCAN, GASA, FCM, and K-means. To verify the operational efficiency of the proposed method, the computation times are calculated and compared. All simulations were conducted in MATLAB R2021b, using an Intel Core i7-7500U CPU of 2.70 GHz and 8 GB of 2133 MHz DDR4 RAM. As shown in Table 2, the computation time for GASA was 14.96 s, while the computation time for the CYYM algorithm was 4.5392 s, indicating that the computational time is one-third of the original. In the CYYM algorithm, $J_{b}$ is the objective function used to search for the fitness value, and $J_{b}$ = 0.1244. This approach greatly improves the precision of the estimated matrix.

**Figure 12:**
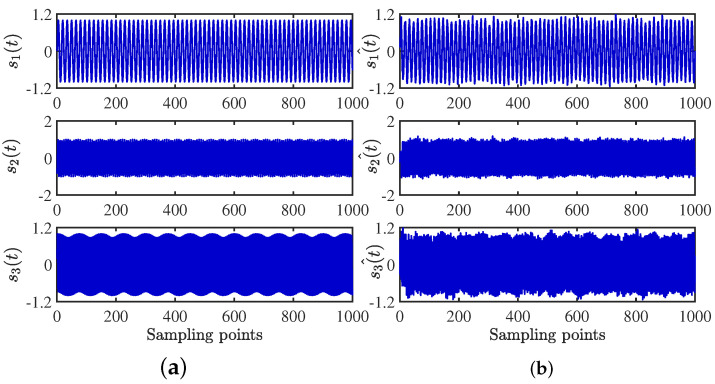
Time− domain signal comparison diagram: (**a**) source signals; (**b**) recovery Signal.

**Figure 13:**
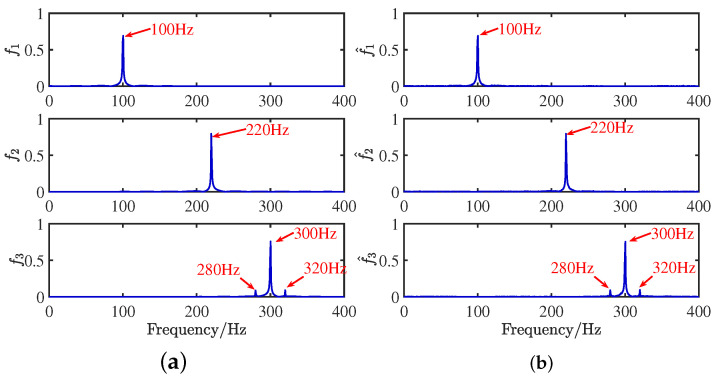
Frequency domain signal comparison diagram: (**a**) source signals; (**b**) recovery signal.

After obtaining the estimated matrix, the shortest path method is used to recover the source signal [29]. For the length limitation, only the source signal and the signal recovered by the CYYM method are given. To better show the superiority, the source signals and the separated signals are shown in Figure 12. Moreover, their Fourier spectrums are shown in Figure 13. For comparison, we find that the three separated signals are consistent with the source signal graph, which indicates that the source signal can be recovered well by the proposed method.

### 4.3. Simulation Experiment 2: Comparative Evaluation of Signal Recovery

**Figure 14:**
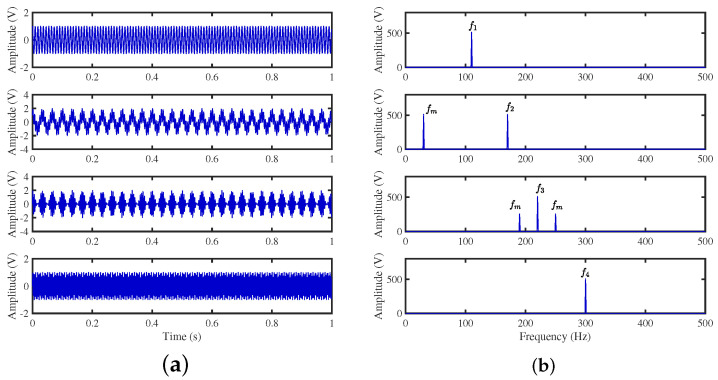
Source signals: (**a**) Waveforms. (**b**) Fourier spectrums.

**Figure 15:**
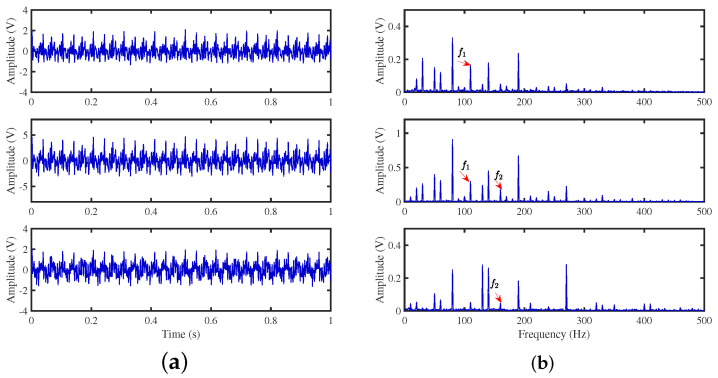
Mixed signals: (**a**) waveforms; (**b**) Fourier spectra.

To achieve the estimation of the mixing matrix and the recovery of source signals, we employed the TIFROM and DEMIX methods, along with the traditional clustering approach DBSCAN, in conjunction with the method proposed in this paper. Through simulated experiments, we mixed four distinct mechanical vibration signals, denoted as $S={\left[s_{1},s_{2},s_{3},s_{4}\right]}^{T}$, into three composite signals. The source signals are depicted in Figure 14. Specifically, $s_{1}$ represents a low-frequency signal and $s_{2}$ corresponds to a frequency-modulated (FM) signal with a carrier frequency of $f_{2}$ and a modulation frequency of $f_{m}$. Similarly, $s_{3}$ denotes an amplitude-modulated (AM) signal with a carrier frequency of $f_{3}$ and a modulation frequency of $f_{m}$. Lastly, $s_{4}$ is characterized as a high-frequency signal, shown in Equation (29). The sampling frequency is f = 1024 Hz and a sampling time of 1 s. The mixed signals are depicted in Figure 15.

(29) $$\begin{array}{c} \left\{\begin{array}{c} s_{1}=\cos\left(2\pi f_{1}t\right);\\ s_{2}=\sin\left(2\pi f_{2}t\right)+\cos\left(2\pi f_{m}t\right);\\ s_{3}=\left(\cos\left(2\pi f_{m}t\right)+1\right)\sin\left(2\pi f_{3}t\right);\\ s_{4}=\sin\left(2\pi f_{4}t\right). \end{array}\right. \end{array}$$

where $f_{1}$ = 110 Hz, $f_{2}$ = 170 Hz, $f_{3}$ = 220 Hz, $f_{4}$ = 300 Hz, and $f_{m}$ = 30 Hz. In MATLAB, a random matrix A is generated by the function generator, as shown in Equation (30):

(30) $$A=\left[\begin{array}{cccc} 0.3874 & 0.3090 & 0.4388 & 0.2040\\ 0.8951 & 0.8103 & 0.8687 & 0.7744\\ 0.1948 & 0.4952 & 0.1883 & 0.5487 \end{array}\right]$$

Gaussian white noise with a mean of 0 and a variance of 0.1 is added to the mixed signal X(t) to simulate real environmental noise, as shown in Equation (31):

(31) X(t)=A×S(t)+0.1×randn(3,N)

**Figure 16:**
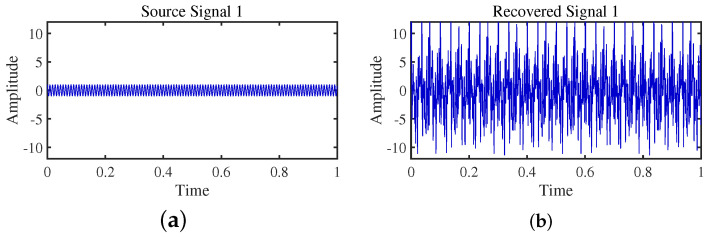
Time-domain signal: (**a**) Source signal $s_{1}$. (**b**) $s_{1}$ obtained by TIFROM method.

**Figure 17:**
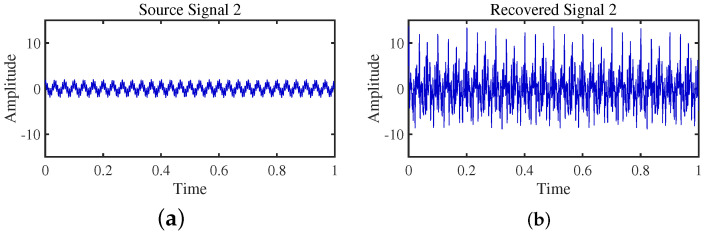
Time-domain signal: (**a**) Source signal $s_{2}$. (**b**) $s_{2}$ obtained by TIFROM method.

**Figure 18:**
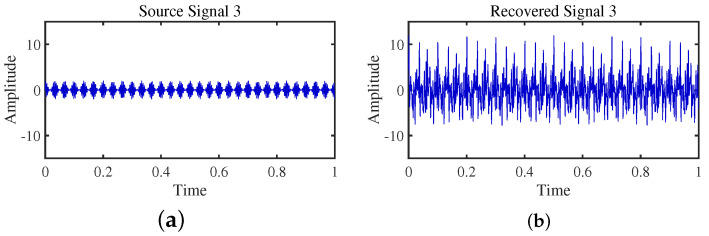
Time-domain signal: (**a**) Source signal $s_{3}$. (**b**) $s_{3}$ obtained by TIFROM method.

**Figure 19:**
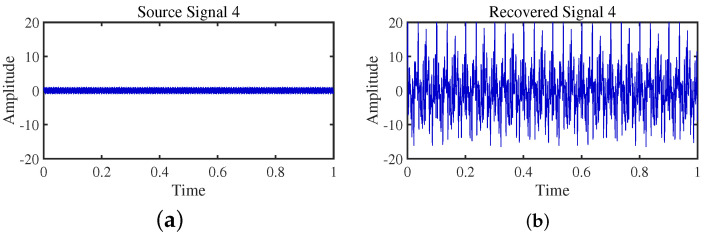
Time -domain signal: (**a**) Source signal $s_{4}$. (**b**) $s_{4}$ obtained by TIFROM method.

The TIFROM algorithm (Time–Frequency Ratio of Mixtures) is designed for blind source separation, aiming to enhance the extraction of independent components through a temporal recurrent structure and an orthogonalization mechanism. However, in simulation experiments, the algorithm exhibits notable shortcomings, as shown in Figure 16, Figure 17, Figure 18 and Figure 19.

Firstly, the TIFROM algorithm demonstrates a significant issue of severe amplitude distortion in signal recovery. This may be attributed to the algorithm’s inability to effectively preserve the amplitude information of the original signals, resulting in noticeable differences in amplitude between the separated signals and the actual signals.

Secondly, a lack of matching in graphical representation is another issue affecting the performance in simulation experiments. The TIFROM algorithm might introduce errors during the operations of the temporal recurrent structure and orthogonalization mechanism, causing the separated signals to deviate from the actual signals in terms of shape. This can hinder the accurate reflection of the original signals’ temporal characteristics in the separated signals.

Additionally, the low precision of the generated mixing matrix by the TIFROM algorithm, as evidenced by the first column angle deviations of 18.5564 and 18.3191, as shown in Table 3, can impact the accuracy of estimating the mixing process during blind source separation.

**Figure 20:**
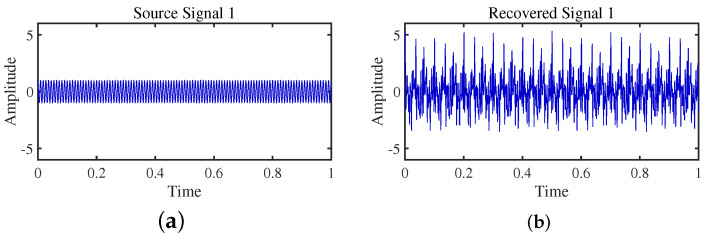
Time-domain signal: (**a**) Source signal $s_{1}$. (**b**) $s_{1}$ obtained by DEMIX method.

**Figure 21:**
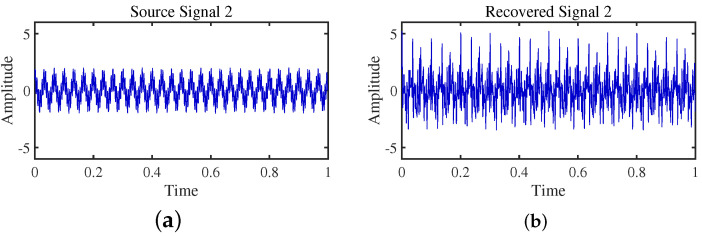
Time -domain signal: (**a**) Source signal $s_{2}$. (**b**) $s_{2}$ obtained by DEMIX method.

**Figure 22:**
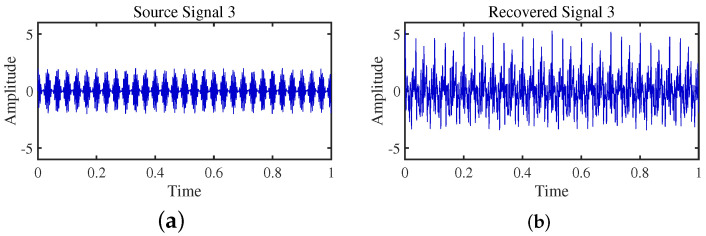
Time-domain signal: (**a**) Source signal $s_{3}$. (**b**) $s_{3}$ obtained by DEMIX method.

**Figure 23:**
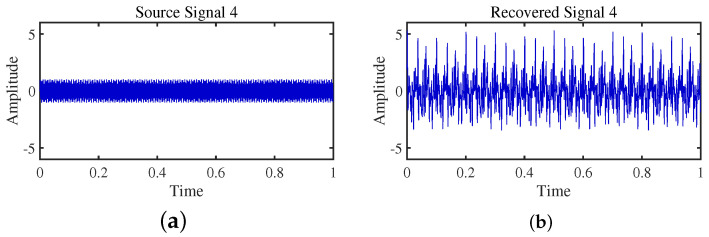
Time-domain signal: (**a**) Source signal $s_{4}$. (**b**) $s_{4}$ obtained by DEMIX method.

**Figure 24:**
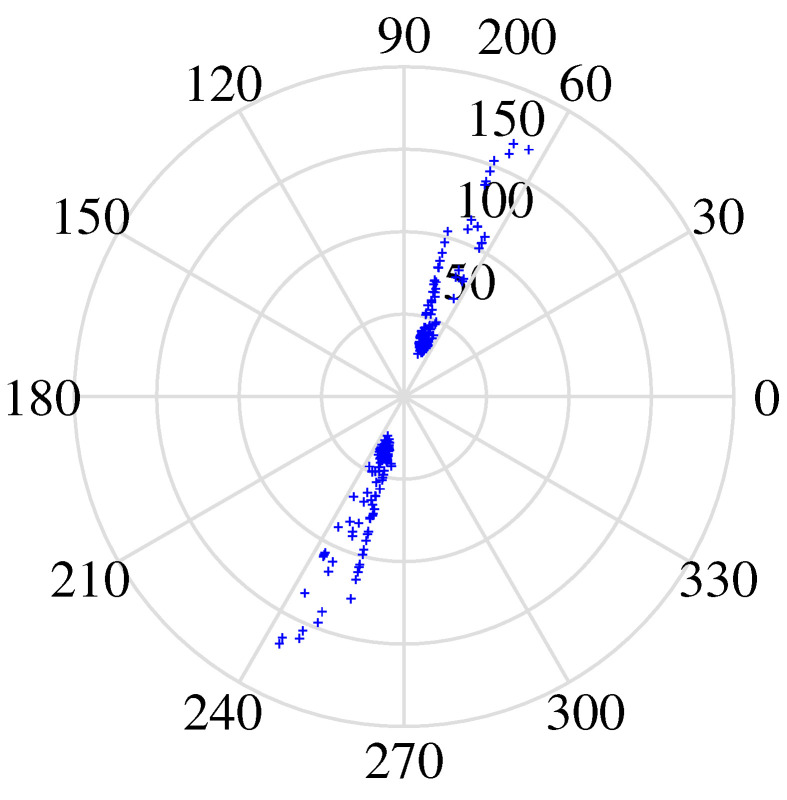
Number of clusters performance map.

DEMIX (Direction Estimation of Mixing matrix) employs a clustering algorithm that prioritizes reliable time–frequency regions, leveraging a local confidence measure. In Table 3, despite a larger angle deviation in the second column (4.3438), DEMIX excels in signal recovery, showcasing its effectiveness in extracting source signals from complex mixtures. The algorithm demonstrates precision in estimating the mixing matrix, forming a robust foundation for separation. In Figure 20, Figure 21, Figure 22 and Figure 23, a notable limitation is the potential inaccuracy in amplitude reconstruction, leading to distortions in signal strength. The graphical representation of separated signals may slightly deviate, impacting accurate signal shape representation. Despite strengths, DEMIX encounters challenges in estimating cluster numbers, illustrated in Figure 24. The graphical representation, while informative, poses challenges in precisely discerning cluster counts due to the convergence of lines.

**Figure 25:**
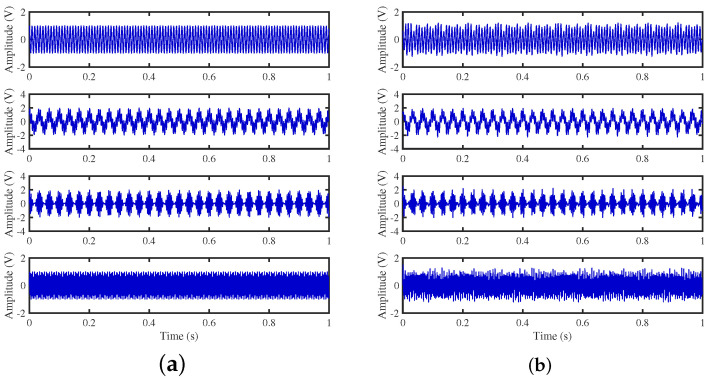
Time-domain signal: (**a**) Source signals. (**b**) Estimated signals obtained by CYYM method.

The signals recovered by the CYYM method are illustrated in Figure 25. The waveforms exhibit similarity, and the amplitudes are accurately reproduced. As shown in Table 3, the minimum NMSE value is −44.1980, and the angular deviations in each column are relatively small. There is no prominent issue of excessively large angle deviations in any column, as observed in the DEMIX method. Through in-depth comparisons with other advanced methods such as TIFROM and DEMIX, we aim to gain a more comprehensive understanding of the strengths and limitations of various approaches, driving progress in the field of blind source separation.

### 4.4. Experiment 3: Compression Machine Trials and Comparative Analysis of Anti-Noise Performance

**Figure 26:**
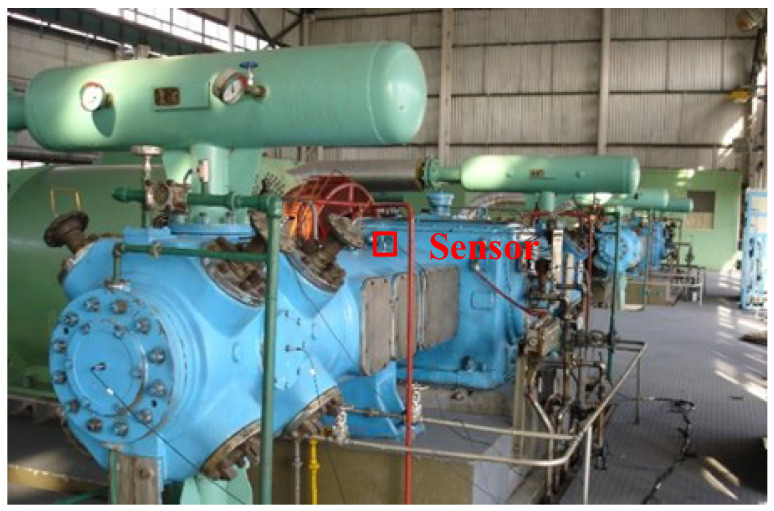
DW-10/12-27-Xlll type two-stage double-acting reciprocating compressor.

**Figure 27:**
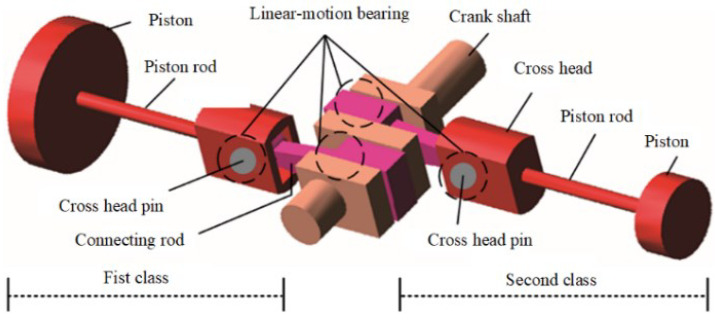
The driving schematic of the compressor mechanism.

**Figure 28:**
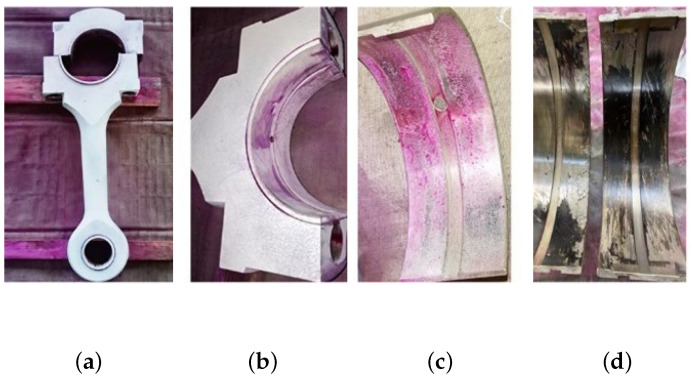
Composition of the reciprocating compressor connecting rod: (**a**) connecting rod; (**b**) big head of the connecting rod; (**c**) bearing bush; (**d**) failure bearing bush.

The two-stage double-acting reciprocating compressor is illustrated in Figure 26. The structural parameters are listed in Table 4, and its model number is DW-10/12-27-Xlll. The driving schematic of the reciprocating compressor mechanism is shown in Figure 27. The connecting rod becomes more fragile and operates under alternating stress. The composition of the reciprocating compressor connecting rod is shown in Figure 28.

To obtain the vibration state information of the connecting rod, the sensor is fixed on the surface of the compressor shell close to the first cross head in Figure 28 using magnetic suction. The corresponding vibration data of three states (normal state $s_{1}$, big end fault state $s_{2}$, and small end fault state $s_{3}$) are collected. In this experiment, we used a multichannel intelligent data acquisition instrument and an ICP accelerometer for the data acquisition system. The sensitivity is 100 mpg, the range is −50∼+50 g, and the frequency range is 0.5∼5 kHz. The reciprocating compressor motor’s characteristic frequency is 8.27 Hz. The sampling frequency is set to 50 kHz. Each state’s signal acquisition time lasts four seconds. To reduce the computation, this paper only selects the first 0.2 s vibration signal for analysis, the corresponding data length of 10,000 points.

**Figure 29:**
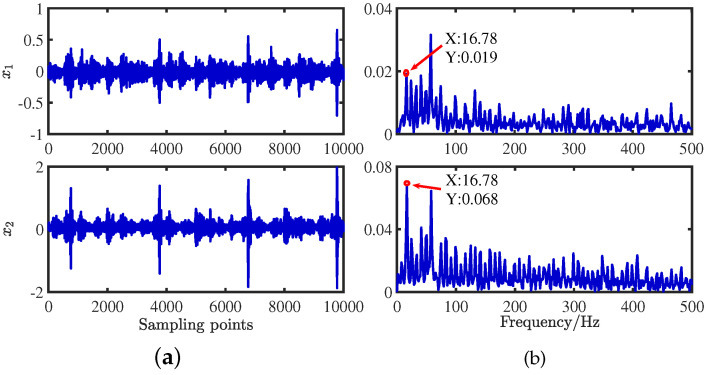
Mixed signals: (**a**) time−domain waveforms; (**b**) envelope spectra.

The three sampled source signals $S={\left[s_{1},s_{2},s_{3}\right]}^{T}$ are thoroughly mixed using a random matrix A of 2 × 3, and the mixed signal $X={\left[x_{1},x_{2}\right]}^{T}$ is obtained in Figure 29. A random mixing matrix is generated randomly by the MATLAB command, shown in Equation (32):

(32) $$A=\left[\begin{array}{ccc} 0.9695 & 0.3235 & 0.3948\\ 0.2452 & -0.9462 & 0.9188 \end{array}\right]$$

The three signal mixing systems measured are shown in Equation (33):

(33) X(t)=A×S(t)

**Figure 30:**
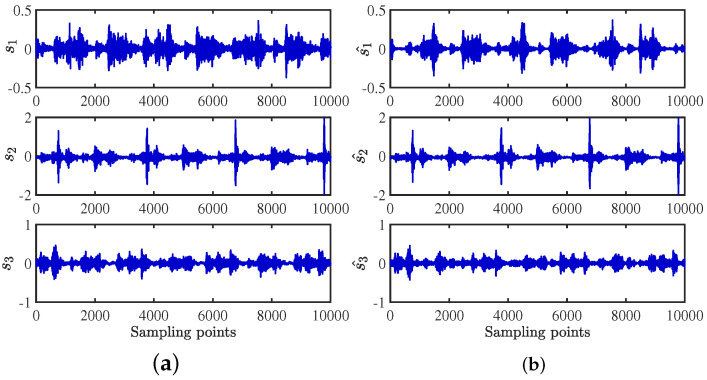
Time-domain contrast diagram of compressor signals: (**a**) source signals; (**b**) recovery signals by the CYYM method.

**Figure 31:**
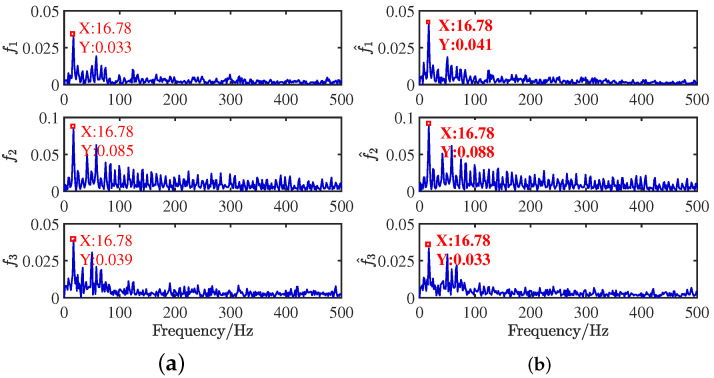
Frequency domain contrast diagram of compressor signals: (**a**) source signals; (**b**) recovery signals by the CYYM method.

The source signals are recovered by the shortest path method [14]. When the source signals are compared with the recovered compressor signals in the time domain, as shown in Figure 30, it is apparent that the result is satisfactory. The frequency distributions are almost identical in Figure 31. The critical information of dual frequency is accurately displayed, and the separation effect is ideal.

In the context of Compression Algorithm Validation Experiments, the accuracy of estimating the mixed matrix is evaluated using the Normalized Mean Squared Error (NMSE). To further assess the similarity between the separated signals and the source signals, we have introduced the correlation coefficient. The comparison of mean results from 100 compression experiments, conducted using six different methods (k-means, FCM, DBSCAN, ADBSCAN, GASA, CYYM), is presented in Table 5. It is evident that the K-means algorithm exhibits the poorest performance in terms of both correlation coefficients and NMSE. On the other hand, FCM, GASAN, DBSCAN, and ADBSCAN yield comparable results. The CYYM method stands out with the lowest NMSE value, recording an impressive −38.962, demonstrating its clear superiority over other algorithms. Additionally, the adaptive DBSCAN algorithm excels in two specific correlation coefficient aspects when compared to other algorithms, with NMSE results slightly favoring DBSCAN. Furthermore, to validate the operational efficiency of the proposed method, running times are calculated and compared. Table 6 indicates that GASA took 22.8614 s, whereas CYYM only took 8.3911 s, which is nearly a third of SAGA’s time. These findings suggest that the improved GASA algorithm enhances the calculation efficiency by appropriately setting the parameters.

### 4.5. Comparative Performance Analysis: NMSE, Correlation Coefficient, and SIR under Varying Signal-to-Noise Ratios

To simulate the noise, we employed a Gaussian noise generation method. The root mean square (RMS) standard deviation of the noise was controlled by the signal-to-noise ratios (SNRs) of 10 dB, 15 dB, 20 dB, 25 dB, and 30 dB to the compressed signals within the compressor. This approach allowed us to introduce noise of varying intensity under different SNR conditions. Specifically, the RMS standard deviation of the noise was calculated as follows:

(34) $$\sigma_{noise}=\sqrt{\frac{\sum \sum {\left(A\times S\right)}^{2}}{\left(2\times N\right)\times 10^{\frac{SNR_{dB}}{10}}}}$$

where $SNR_{dB}$ represents the signal-to-noise ratio in decibels, *A* is the signal matrix, *S* is the source signal, and *N* is the signal length. This noise generation method played a pivotal role in facilitating the simulation of signal interference and noise across a range of SNR conditions during our three experiments. In each of these three experiments, we meticulously calculated essential metrics, encompassing the Normalized Mean Squared Error (NMSE), correlation coefficients, and Signal-to-Interference Ratio (SIR). It is noteworthy that each experiment maintained a consistent mixed system configuration, and this setup was subjected to 100 repetitions as part of our Monte Carlo analysis. Subsequently, we computed the mean values of these metrics. This stringent methodology afforded us a comprehensive evaluation of matrix estimation accuracy and the precision of signal recovery, spanning a diverse array of noise levels.

**Figure 32:**
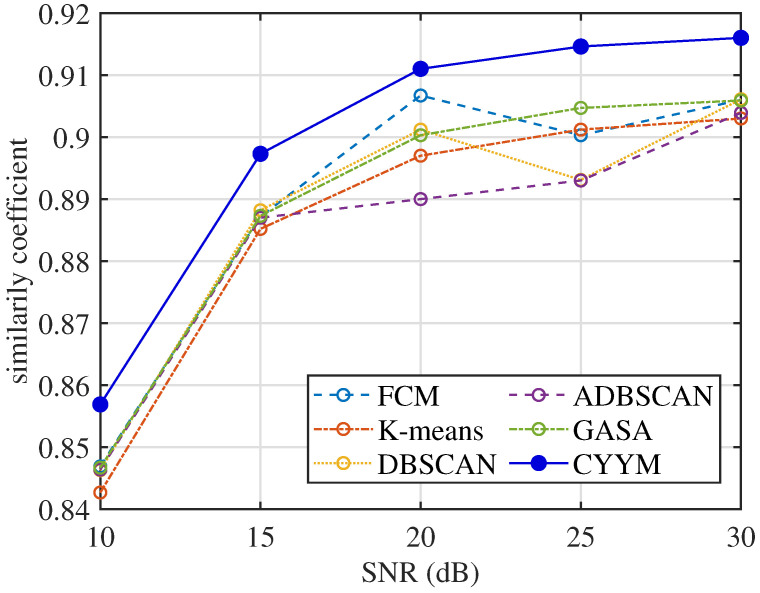
Comparison diagram of correlation coefficients.

From the comparative analysis of correlation coefficients at varying signal-to-noise ratios (SNRs), in Figure 32, it is evident that all methods consistently exhibit correlation coefficients exceeding 0.84, indicating a high level of signal recovery accuracy. However, it is noteworthy that the correlation coefficients of the other five methods exhibit erratic fluctuations as SNR increases, demonstrating their instability under changing noise conditions. In contrast, the method employed in this study demonstrates a stable and consistently increasing correlation coefficient with the increment of SNR, underscoring its robustness in noisy environments.

**Figure 33:**
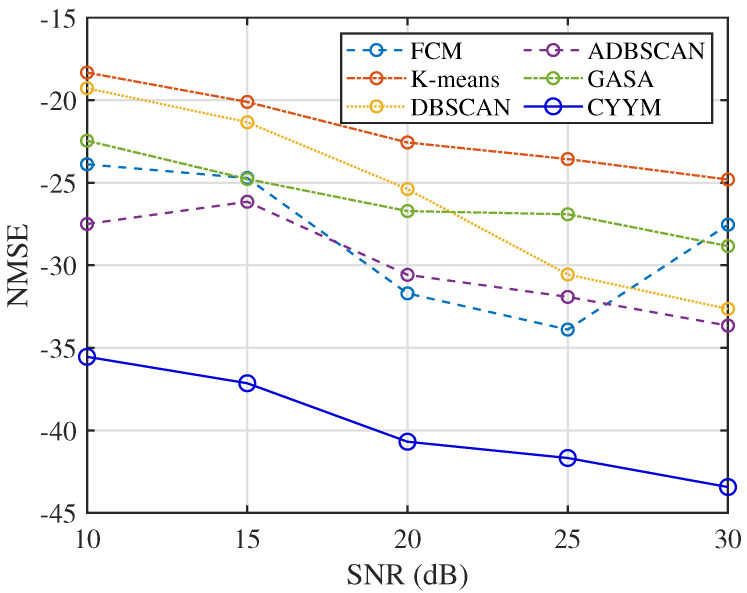
Comparison diagram of NMSE.

In terms of the accuracy of mixed matrix estimation in Figure 33, the other five methods exhibit relatively similar performance. In contrast, the method employed in this study demonstrates improved performance with decreasing NMSE values as SNR increases. This observation underscores the method’s strong adaptability to noise conditions and its enhanced precision in mixed matrix estimation.

**Figure 34:**
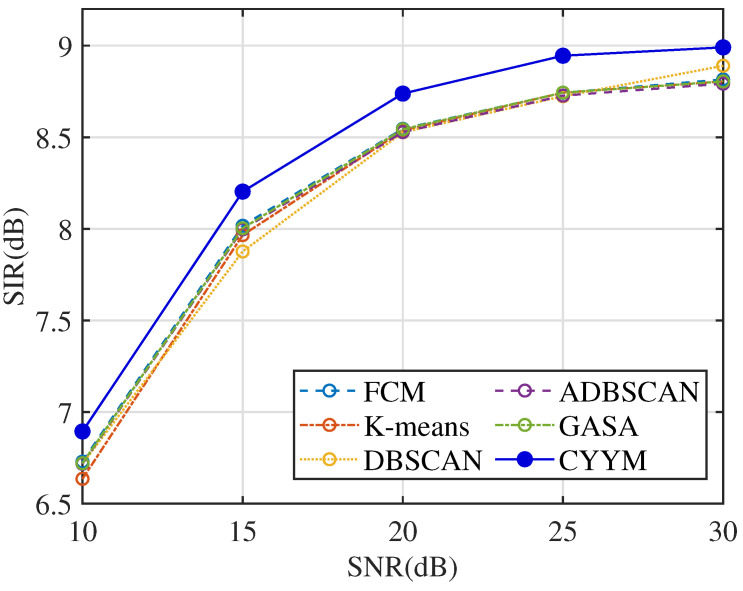
Comparison diagram of SIR.

SIR is employed to assess the relative strength between the target vibration signal and noise. Higher SIR values facilitate the extraction of fault signals from complex compressor vibration data. Observing the SIR comparison across different signal-to-noise ratios in Figure 34, it becomes evident that the mean SIR values for all six methods exhibit a robust increase, although they remain below 10. Notably, Table 7 highlights that the SIR indicator for the detection of major crankshaft faults exceeds the threshold of 11, indicating the superior performance of the method employed in this study in extracting major fault signals. This outcome further underscores the significance of parameter selection and adjustment in enhancing the separability of major fault signals.

### 4.6. Compressor Fault Detection

In the context of rolling bearings, fault diagnosis primarily relies on signal spectrum analysis. However, when it comes to compressors, achieving fault type and location determination through simple spectrum analysis is often challenging. This challenge arises from the fact that faults occurring at different locations within the compressor generate signals with identical frequencies, as illustrated in Figure 31. Consequently, using recovered signals alone to identify faults poses a significant challenge. To address this, efforts have been dedicated to utilizing entropy as a quantitative measure for further characterizing the vibration signal’s fault-related attributes.

Superior to most nonlinear dynamic measures such as Sample entropy and Multi-scale fuzzy entropy, Refined composite multiscale fuzzy entropy (RCMFE) has higher accuracy of entropy estimation and can reflect the fault state characteristics more comprehensively. The regularity of signal entropy varies when different faults occur in the compressor, and can, thus, be used as a characteristic feature of compressor signals for the faults. After long-term monitoring, our laboratory obtained a library of compressor fault characteristics and recorded the corresponding characteristic shape curves. The better the distinguishing result of different faults is, the more effective the method is for fault classification. The higher the similarity between the estimated signal and the entropy curve of the fault library, the more efficient the method is in determining faults.

**Figure 35:**
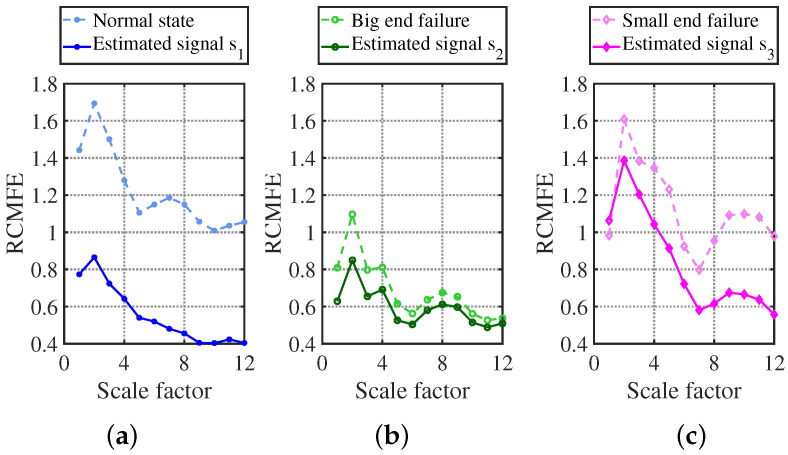
RCMFE characterization curve vs. fault library identification plot: (**a**) normal state; (**b**) big end failure; (**c**) small end failure.

RCMFE has excellent characteristic results, as shown in Figure 35. The three states’ entropy characteristic curves are blue, green, and pink dashed lines, which can be completely distinguished by the naked eye due to the vast difference in shape characteristics, and the three states are normal state, first-stage connecting rod large head failure, and first-stage connecting rod small head failure.

The graph of the signal estimated by the CYYM method is shown in Figure 18a with a dark blue solid line. From the graph, it can be found that as the scale factor increases, the dashed line and the solid line change in a highly consistent trend, and the recovered signal has better stability because of the filtered noise. Thus, the overall entropy value decreases, indicating that the dark blue solid line signal is the normal state compressor signal. Meanwhile, the dark green solid line after the restoration with CYYM is shown in Figure 18b. The two green curved lines are highly similar in shape, from which it can be determined that the signal fault is a first-level connecting rod large head fault; in Figure 18c, the light pink dashed line is the graph of the entropy value of the first-level connecting rod small head fault in the fault library, while the entropy value of the restored signal is shown in Figure 18c with a dark pink solid line. Despite the two graphs being slightly different, the huge decline of the entropy value from the highest point to the lowest point and the appearance of the wave after the scale factor being greater than 7 indicate that the two fault characteristics are identical.

## 5. Conclusions

In this study, our goal was to address the limitations of the traditional clustering algorithm FCM, which requires prior knowledge to determine the number of signal sources and is prone to getting stuck in local optima. To overcome these challenges, we introduced the GASA optimization method with adaptive DBSCAN clustering initialization as a novel approach for accurately estimating underdetermined mixing matrices. The implemented CYYM method in this research demonstrated significant capabilities. It not only automatically predicted the number of sources by adaptively adjusting DBSCAN parameters, but also achieved precise localization of clustering centers. Furthermore, the application of the CYYM method in diagnosing compressor connecting rod faults significantly improved search and evolution speeds compared to the GASA algorithm. The combination of the CYYM method with Refined Composite Multiscale Fuzzy Entropy (RCMFE) analysis successfully achieved fault diagnosis, identifying fault types and their locations.

In this study, our focus shifted to improving the estimation of mixing matrices in the case of instantaneous mixtures. Despite making substantial progress in fault diagnosis, it is crucial to acknowledge that this method is not suitable for estimating mixing matrices in situations involving delays.

Additionally, the current fault feature classification involves a relatively limited sample size of single faults. Future work should concentrate on expanding the RCMFE fault dataset by collecting field measurements of more typical faults. Moreover, exploring the application of intelligent algorithms, such as neural networks, can enhance fault classification capabilities [52].

This study lays the groundwork for further research in the field, with the potential to enhance fault detection and classification methodologies.

## Figures and Tables

**Table 1:** Comparisons of angular differences and NMSE metrics.

Method	Angular Difference			NMSE (dB)
	$\mathrm{ang}(a_{1},{\hat{a}}_{1})$	$\mathrm{ang}(a_{2},{\hat{a}}_{2})$	$\mathrm{ang}(a_{3},{\hat{a}}_{3})$	
Kmeans	0.4100	1.1868	0.3226	−38.4100
FCM	0.2639	0.3040	0.2246	−46.4680
GASA	0.3170	0.1234	0.1347	−48.5710
DBSCAN	0.1039	0.1661	0.1659	−51.7364
ADBSCAN	0.0093	0.1074	0.0093	−59.1250
CYYM	**0.0001**	**0.0016**	**0.0032**	**−74.1040**

**Table 2:** Running time of different methods.

Method	GASA	The Proposed Method
Running time	14.96 s	4.539 s

**Table 3:** Comparisons of angular differences and NMSE metrics.

Method	Angular Difference				NMSE (dB)
	$\mathrm{ang}(a_{1},{\hat{a}}_{1})$	$\mathrm{ang}(a_{2},{\hat{a}}_{2})$	$\mathrm{ang}(a_{3},{\hat{a}}_{3})$	$\mathrm{ang}(a_{4},{\hat{a}}_{4})$	
TIFROM	**18.5564**	**18.3191**	0.0587	0.0167	−7.9891
DEMIX	0.0023	**4.3438**	0.0041	0.0021	−36.1021
DBSCAN	0.5555	0.8192	0.4062	1.0298	−33.9479
CYYM	0.0530	0.0043	0.0228	0.5943	**−44.1980**

**Table 4:** The structural parameters of the reciprocating compressor.

Shaft Power	Piston Stroke	Crankshaft Speed
500 kW	240 mm	496 rpm

**Table 5:** Correlation coefficients and NMSE.

Methods	Correlation Coefficient R			NMSE (dB)
	$\langle s_{1},{\hat{s}}_{1}\rangle$	$\langle s_{2},{\hat{s}}_{2}\rangle$	$\langle s_{3},{\hat{s}}_{3}\rangle$	
Kmeans	0.8519	0.9770	0.8881	−23.8561
DBSCAN	0.8560	0.9766	0.8879	−26.4720
ADBSCAN	0.8540	**0.9768**	0.8878	−28.2293
FCM	0.8544	0.9769	0.8878	−28.7745
GASA	0.8758	0.9698	0.8207	−30.5559
CYYM	**0.8809**	0.9706	**0.8976**	**−38.9623**

**Table 6:** Running time of comparison.

Method	GASA	Proposed Method
Running time	28.8614 s	8.3911 s

**Table 7:** SIR indicator for the detection of major crankshaft faults.

SNR	10 db	15 db	20 db	25 db	30 db
SIR	11.4748	12.9818	13.6118	13.9570	14.0181

## Data Availability

The datasets used or analyzed during the current study are available from the corresponding author on reasonable request.
